# Correction: A Neolithic Mega-Tsunami Event in the Eastern Mediterranean: Prehistoric Settlement Vulnerability Along the Carmel Coast, Israel

**DOI:** 10.1371/journal.pone.0247953

**Published:** 2021-02-25

**Authors:** Gilad Shtienberg, Assaf Yasur-Landau, Richard D. Norris, Michael Lazar, Tammy M. Rittenour, Anthony Tamberino, Omri Gadol, Katrina Cantu, Ehud Arkin-Shalev, Steven N. Ward, Thomas E. Levy

There is an error in reference 18. The correct reference is: Sivan, D., Potasman, M., Almogi-Labin, A., Bar-Yosef Mayer, D.E., Spanier, E., Boaretto, E., 2006. The Glycymeris query along the coast and shallow shelf of Israel, southeast Mediterranean. Palaeogeogr. Palaeoclimatol. Palaeoecol. 233, 134–148.

There is an error in reference 31. The correct reference is: Braun, Y., Kagan, E., Bar-Matthews, M., Ayalon, A., Agnon, A., 2011. Dating speleoseismites near the Dead Sea Transform and the Carmel Fault: Clues to coupling of a plate boundary and its branch. Isr. J. of Earth Sci. 58, 257–273.

The sixth sentence of the fifth paragraph in the Results should have cited reference 36 instead of 35. As a result, all subsequent references are misnumbered. References 35–47 should be references 36–48.

There is an error in the caption for Fig 3, “Core analysis and chronostratigraphic correlation.” Please see the complete, correct Fig 3 caption here.

**Fig 3:**
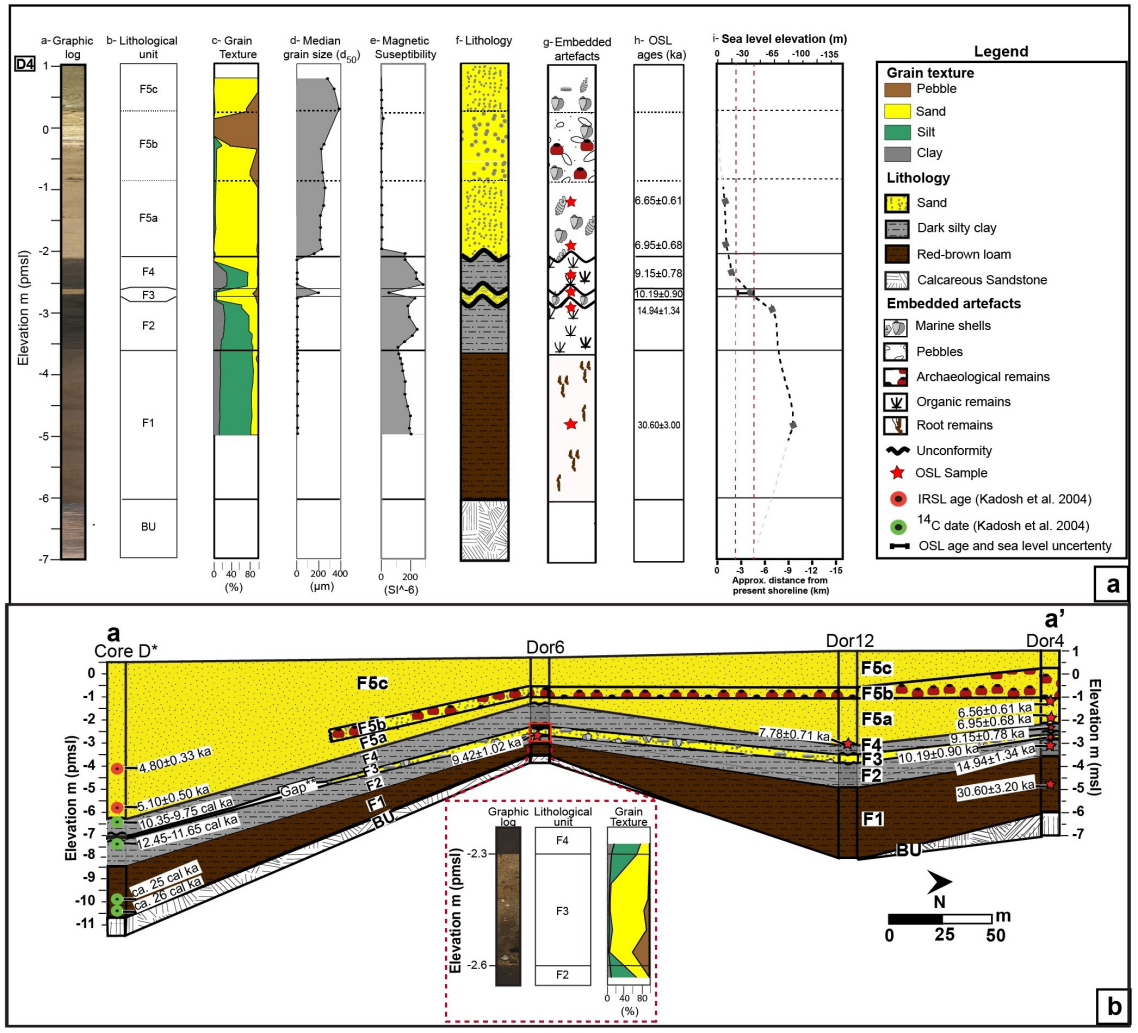
Core analysis and chronostratigraphic correlation. (a) Borehole D4 with core scan; lithological unit name; sedimentological and petrophysical results; lithological identification with OSL sampling location; OSL ages presented before 2018; and corresponding sea level [24,25] as well as approximate shoreline location. (b) Chronostratigraphic cross sections in the coastal area of Dor based on sedimentological and OSL data obtained in the present study, presented for thousand years ago (ka, marked with a red star) as well as shtienberg et al. (submitted; blue star) correlated with the lithological results, and 14C calibrated dates (cal. ka; green circles) published in Kadosh et al. [16]. A closeup of units F2, F3 and F4 in core D6 with its lithological unit name and accumulative grain texture results. The modern topography portrayed in the cross sections was extracted from the DEM presented in Fig 2B. See Fig 2B for cross section location.
