# Supplementary material for: A Neolithic mega-tsunami event in the eastern Mediterranean: Prehistoric settlement vulnerability along the Carmel coast, Israel
Source: PLoS One. 2020 Dec 23;15(12):e0243619. doi: 10.1371/journal.pone.0243619 (PMC7757801; doi:10.1371/journal.pone.0243619)
Supplement: S3 Fig — (DOCX) [file pone.0243619.s003.docx]

**
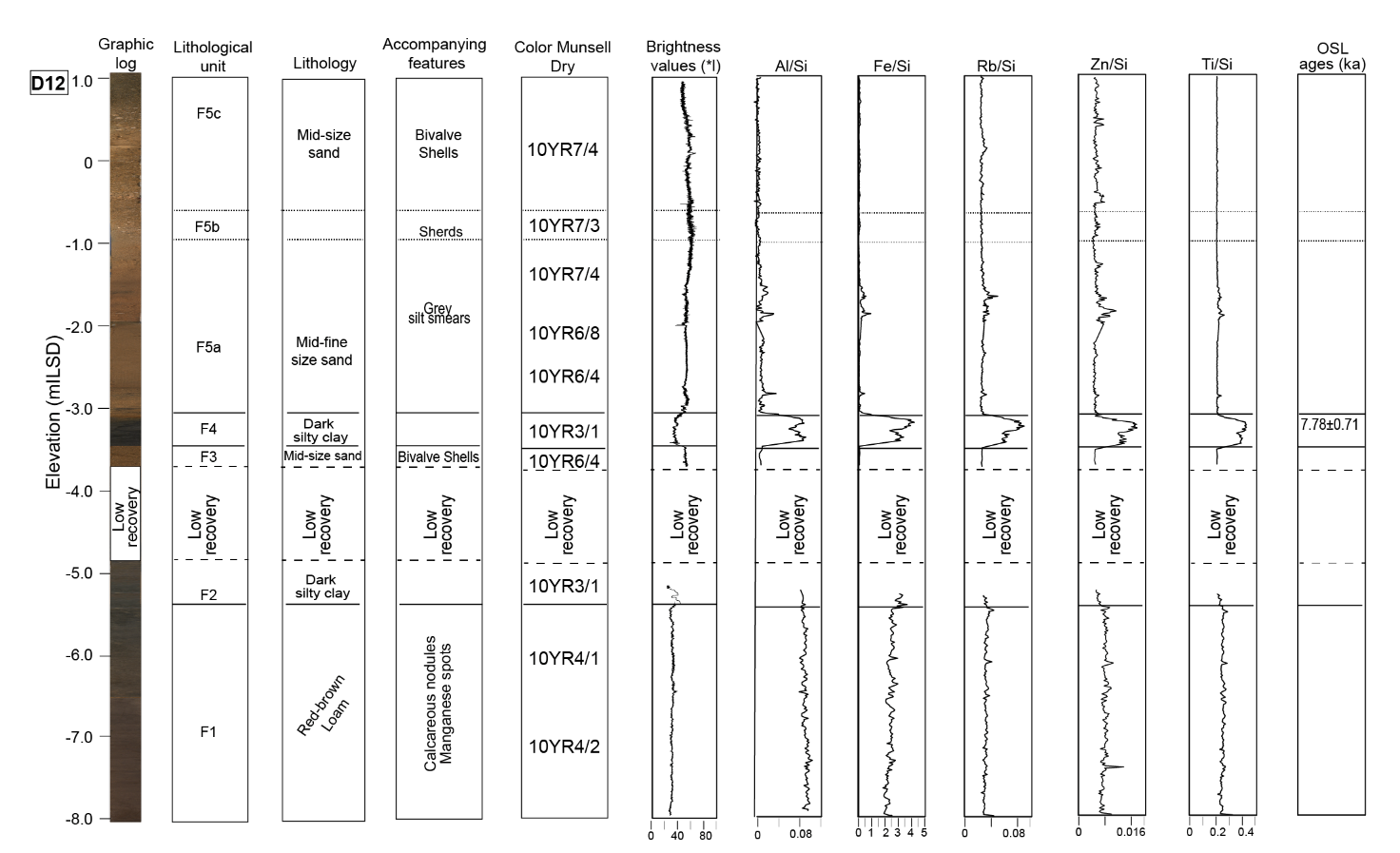
**

**S3 Fig.** Borehole D12 (location is displayed in Fig. 2b) with lithological classification, description, accompanying features, brightness differences, relative elemental concentration variations and OSL data obtained in the present study.
