# Supplementary figures and images for: A Neolithic mega-tsunami event in the eastern Mediterranean: Prehistoric settlement vulnerability along the Carmel coast, Israel
Source: PLoS One. 2020 Dec 23;15(12):e0243619. doi: 10.1371/journal.pone.0243619 (PMC7757801; doi:10.1371/journal.pone.0243619)

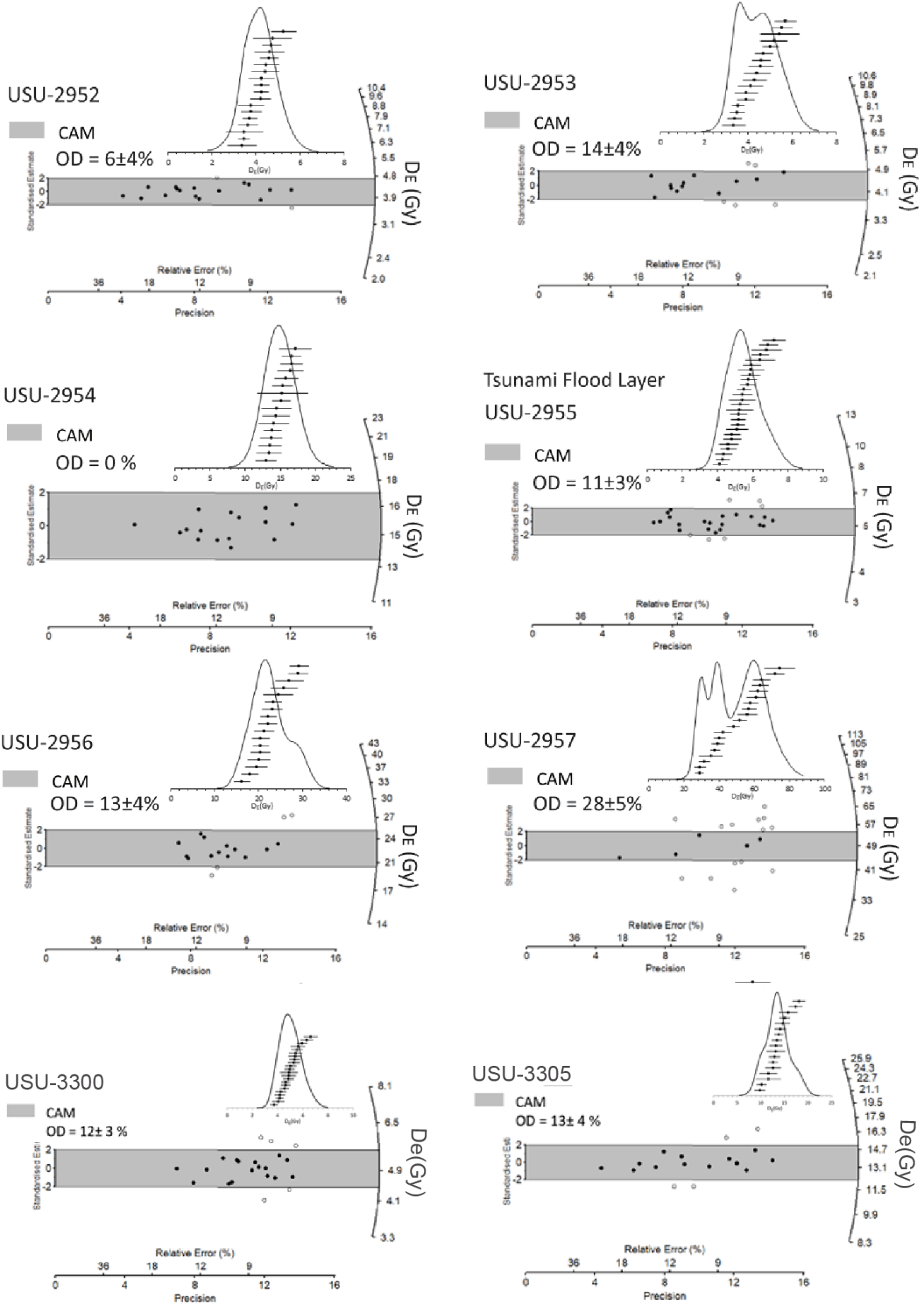


**S4 Fig.** Equivalent Dose distributions for OSL samples. CAM = Central Age Model. OD = Over-dispersion

Supplement: S4 Fig — CAM = Central Age Model. OD = Over-dispersion. (DOCX) [file pone.0243619.s004.docx]
