# Supplementary material for: A Neolithic mega-tsunami event in the eastern Mediterranean: Prehistoric settlement vulnerability along the Carmel coast, Israel
Source: PLoS One. 2020 Dec 23;15(12):e0243619. doi: 10.1371/journal.pone.0243619 (PMC7757801; doi:10.1371/journal.pone.0243619)
Supplement: S1 Table — The location of these events and deposits is annotated in Fig 1. Ages with * are ones that are presented for (2σ). (DOCX) [file pone.0243619.s005.docx]

**S1 Table.** Compilation of previously dated tsunami deposits occurring along the eastern Mediterranean coast. The location of these events and deposits is annotated in Figure 1. Ages with * are ones that are presented for (2σ).

| Reference number on map | Country/location | Date of event Anno Domini (AD)/Before Crist (BC) Calibrated age (cal.) | Approximated age (ka) | Reference |
| --- | --- | --- | --- | --- |
| 1a | Egypt/Alexandria | 365 AD (± 100) | 1.65±100 | 1 |
| 2a | Egypt/Alexandria | 700-899 cal. AD* | 1.31-1.1 | 2 |
| 3a | Cyprus/Paphos, Polis, Cape, Greco | 1354-1950 cal AD* | 0.66-0.07 | 3 |
| 4a | Israel/Caesarea Marittima | 188 cal BC - 305 cal AD* | 2.03-1.71 | 4 |
| 5a | Israel/Caesarea Marittima | 1714-1484 cal BC* | 3.73-3.50 | 5 |
| 6a | Israel/Caesarea Marittima | 500-599 AD (6th century AD – archaeological remains indications) | 1.51-1.42 | 6 |
| 7a | Israel/Caesarea Marittima | 1759 AD (± 100) | 0.26±100 | 7 |
| 8a | Israel/Jiser al-Zarka | 6045 – 5569 cal.BP | 6.04-5.56 | 8 |
| 9a | Lebanon/Byblos, Senani Island | 1952-1675 cal BP* | 1.95-1.67 | 2 |
| 10a | Greece/Elos | 1366-840 cal BC* | 3.38-2.85 | 2 |
| 11a | Greece (Crete)/Gramvousa, Balos,  Falasarna, Mavros, Stomiou, Gramenos, Paleochora | 365 AD (± 100) | 1.65±100 | 9 |
| 12a | Greece/Western Crete | 98 cal BC - 564 cal AD* | 2.11-1.45 | 10 |
| 13a | Greece (Crete)/Palaikastro | 1735-1545 cal BC | 3.75-3.56 | 11 |
| 14a | Greece/Pounta | 1453-1631 cal AD* | 0.56-0.38 | 2 |
| 15a | Greece/Astros (Limni Moustou) | 643-1452 cal AD* | 1.37-0.56 | 2 |
| 16a | Greece/Pylos, Porto Gatea, Archangelos, Elaphonisos | 135-352 cal AD* | 1.88-1.66 | 12 |
| 17a | Greece/Gialova (Limni Divariou) | 1200-1000 cal BC* | 3.21-3.01 | 13 |
| 18a | Greece/Santorini | 1650 AD | 0.36 | 12 |
| 19a | Greece (Crete)/Balos bay | 356 AD | 1.66 | 9 |
| 20a | Greece/Thera | 7th - 8th century cal AD | 1.41-1.31 | 2 |
| 21a | SW Turkey/Dalaman | 1303, 1481, 1741 cal AD | 0.71, 0.57, 0.27 | 14 |
| 22a | SW Turkey/Didim | 1650 cal AD | 0.36 | 15 |
