# Supplementary material for: A Neolithic mega-tsunami event in the eastern Mediterranean: Prehistoric settlement vulnerability along the Carmel coast, Israel
Source: PLoS One. 2020 Dec 23;15(12):e0243619. doi: 10.1371/journal.pone.0243619 (PMC7757801; doi:10.1371/journal.pone.0243619)
Supplement: S2 Table — The location of these events and deposits is annotated in Fig 1. (DOCX) [file pone.0243619.s006.docx]

**S2 Table.** Compilation of previously dated tsunami events occurring in the eastern Mediterranean. The location of these events and deposits is annotated in figure 1.

| Site number in map | Date of event Anno Domini (AD)/Before Crist (BC) | Approximated age (ka) | Country/location | Reference |
| --- | --- | --- | --- | --- |
| 1 | 551AD | 1.46 | Lebanon, Israel, Syria | 16,17,18 |
| 2 | 749AD | 1.27 | levant coast | 19 |
| 3 | 1202AD | 0.81 | Syrian/Lebanon | 17 |
| 4 | 1202AD | 0.81 | Cyprus (S-E Cyprus) | 17 |
| 5 | 1759AD | 0.26 | Levant (Akko) | 17 |
| 6 | 1956AD | 0.06 | Yaffo | 17 |
| 7 | 1222AD | 0.80 | Cyprus (S-E Cyprus) | 19 |
| 8 | 1954AD | 0.06 | Cyprus (S-E Cyprus) | 19 |
| 9 | 1033AD | 0.98 | Levant coast | 20 |
| 10 | 1068AD | 0.95 | Levant coast | 2 |
| 11 | 1068AD | 0.95 | Levant coast | 2 |
| 12 | 20AD | 2.00 | The Nile cone (Egypt) | 21 |
| 13 | 25BC | 2.00 | Lebanon | 21 |
| 14 | 115AD | 1.90 | Levant coast | 21 |
| 15 | 803AD | 1.21 | southern turkey | 21 |
| 16 | 1222AD | 0.80 | Cyprus | 21 |
| 17 | 1546AD | 0.47 | Israel | 21 |
| 18 | 1759AD | 0.26 | Lebanon - Israel | 21 |

>
