# Supplementary material for: A Neolithic mega-tsunami event in the eastern Mediterranean: Prehistoric settlement vulnerability along the Carmel coast, Israel
Source: PLoS One. 2020 Dec 23;15(12):e0243619. doi: 10.1371/journal.pone.0243619 (PMC7757801; doi:10.1371/journal.pone.0243619)
Supplement: S3 Table — (DOCX) [file pone.0243619.s007.docx]

**S3 Table**. Correlative archaeological period based on Kuijt and Goring-Morris [22]

| Archaeological period in Levant | Approximated age (ka) |
| --- | --- |
| Pottery Neolithic | 8.25 – 7.80 |
| Pre pottery Neolithic C | 8.70 – 8.25 |
| Pre pottery Neolithic middle – late B | 10.10 – 8.70 |
| Pre pottery Neolithic Early B | 10.50 – 10.10 |
| Pre pottery Neolithic A | 11.70 – 10.50 |
| Late Natufian | 12.50 – 12.00 |
