# Supplementary material for: A Neolithic mega-tsunami event in the eastern Mediterranean: Prehistoric settlement vulnerability along the Carmel coast, Israel
Source: PLoS One. 2020 Dec 23;15(12):e0243619. doi: 10.1371/journal.pone.0243619 (PMC7757801; doi:10.1371/journal.pone.0243619)
Supplement: S1 File — (DOCX) [file pone.0243619.s009.docx]

Extended Luminescence Methods and Results section

*Optical Measurements*

The SAR technique of Murray and Wintle [23] was used for single-grain and small-aliquot analysis of the samples. Optical measurements were performed on Risø TL/OSL Model DA-20 readers with blue-green light emitting diodes (LED) (470 nm, 40 mW/cm^2^) as the stimulation source for small-aliquot measurements at 125ºC through 7.5-mm UV filters (U-340). The signals were calculated by subtracting the average of the last 5 seconds (background signal) from the first 0.7s (4 channels) of the 40s signal decay curve for the small aliquot analyses. Dose response curves were fit within saturating exponential curves to calculate equivalent dose (D_E_) values.

*Equivalent Dose (D_E_) and Error Calculation*

D_E_ distributions from samples show largely symmetric distributions with over-dispersion (OD, scatter) below the 20% cut-off for scatter beyond instrumental error in all but one sample. This sample was from an environment that would be expected to have partial bleaching and mixing and include a sample from the top of the buried soil (USU-2957) (Fig. S4). D_E_ values were calculated using the central age model (CAM) for all samples. Aliquots were rejected if they had evidence of feldspar contamination, recycling ratio <0.1 or >1.1, recuperation >10% of the natural signal, or natural D_E_ greater than the highest regenerative dose given. Errors on D_E_ values are reported at 2-sigma standard error and age estimates are reported at 1-sigma standard error (see Table 1). Uncertainties include errors related to instrument calibration, and dose rate and equivalent dose calculations and were calculated in quadrature using the methods of Guérin et al. [24] and Aitken and Alldred [25].
