# Supplementary material for: A Neolithic mega-tsunami event in the eastern Mediterranean: Prehistoric settlement vulnerability along the Carmel coast, Israel
Source: PLoS One. 2020 Dec 23;15(12):e0243619. doi: 10.1371/journal.pone.0243619 (PMC7757801; doi:10.1371/journal.pone.0243619)
Supplement: S2 File — (DOCX) [file pone.0243619.s010.docx]

Extended result section

4. Results

4.1 The geomorphology of the study area

The study area consists of a 280 m long sandy pocket beach with a mean width of 230 m from its shoreline to the 4-meter-high sand dune in its backshore. The pocket beach is bordered by an aeolianite headland to the North and a tombolo in its southern part. Both features project ~290 m seawards relative to the shoreline. On the northern headland, archeological structures (wells, slipways), as well as Iron-age urban settlement remains, are evident while on the tombolo’s western section, a rock-cut pool is apparent. The pocket beach elevation ranges from 0 to 3 meters relative to present mean sea level (PMSL) with a slope gradient of 1:48 while the surface elevation of the northern headland and tombolo are +10 and +5 PMSL respectively (Fig. 2).

4.2 The stratigraphy of Dor’s south bay pocket beach

The sedimentary sequence of Dor, which was identified in the south bay pocket beach (Fig. 2b), consists of five lithological units (F1, F2, F3, F4 and F5) that uncomfortably overly the Basal Unit (BU). The lithological classification of each unit was obtained through integration of the morphological and sedimentological database of this study. Unit correlation was based on morphological features, thickness, elevations, litho-stratigraphical relations, sedimentological and geochemical similarities (Fig. S1-S3). The stratigraphic correlation and order of the OSL ages presented in the current study have enabled to reconstruct the chronostratigraphy of the study area:

4.2.1 Basal Unit (BU)

The top ~ 0.8 m of the basal unit was reached in Boreholes D4 and D6 (Figs. 3, S1a, b) whose surface elevations range from ˗6.2 to ˗3.5 PMSL respectively. Brightness consists of values of 65 (on a scale of 0 – 100) while color varies from yellowish white – pale grey (2.5Y8/1 and 7.5YR4/4 on the Munsell color chart). The lithological and geochemical properties of this part of the unit consist of indurated-brittle calcareous cemented to partly cemented sand with varying rations of Ti/Si Zn/Si; Fe/Si, Rb/Si; Al/Si.

Based on the sedimentological characteristics, unit age and the unit’s surface elevation in nearby boreholes (Figs. 2b and S1-S3) BU was identified of as the upper parts of the calcareous sandstone unit.

4.2.2 Unit F1

Unit F1, which overlies BU, was identified in Boreholes D4, D12 and D6, with surface elevations ranging from ˗3.6 to ˗5.3 PMSL and a thickness of 0.5 to 2.8 m (Figs. S1-S3). Brightness and color consist of values of ~40 and dark brown – brown hues respectively (10YR4/1 - 10YR4/4 on the Munsell color chart). The lithological properties of this unit are characterized as a silty loam sediment with a mean median grain size (Mz) of 6 µm that consists of irregularly-shaped hard calcareous cemented sand nodules and manganese spots. The size and abundance of nodules and manganese spots decreases upwards and are absent at the top of the unit. F1 consists of Ti/Si; Zn/Si; Fe/Si, Rb/Si; Al/Si ratios that are substantially higher than its underlying unit (BU), while MS values range from 200 to 150×SI-6 (Fig. 3) and for most parts contain no microfauna or any other biological remains. Based on these characteristics, F1 is interpreted as a palaeosol that was dated in core D4 from the bottom to the middle part of the unit by one OSL age to 30.59 ± 3.20 ka (Tables 1, 2; Fig. 3).

4.2.3 Unit F2

Unit F2 was identified in Boreholes D4, D12 and D6, with surface elevations ranging from ˗2.6 to ˗4.8 PMSL and a thickness of 0.5 to 1.0 m (Figs. S1-S3). Brightness and color are characterized by values of ~45 and dark grey – dark brown hues respectively (10YR3/1 - 10YR3/2 on the Munsell color chart). Lithological properties of this unit consist of a homogenous loam sediment with a Mz of 4.5 µm. F2 comprises brackish microfauna as well as fresh to brackish plant remains (Charophyte, Data set 1) while its elemental composition includes similar Al/Si Ti/Si Zn/Si; Fe/Si, Rb/Si ratios compared to its underlying unit. MS values range from 180×SI-6 in its lower half to ~220×SI-6 in its upper half (Fig. 3).

Based on the petro-sedimentological properties as well as the skeletal remains and faunal features unit F2 was interpreted as a wetland deposit. This facies was dated in core D4 by one OSL age to 14.94 ± 1.34 ka while its surface was dated in core D6 to 9.42 ± 0.85 ka (Tables 1, 2; Fig. 3).

4.2.4 Unit F3

Unit F3 unconformably overlies unit F2 and was identified in Boreholes D4, D12 and D6, with surface elevations ranging from ˗2.6 to ˗3.3 PMSL and thickness of 0.12 to 0.4 m (Figs. S1-S3). The exemplifying brightness and color consist of values of ~65 and yellow – light yellow hues respectively (10YR6/4 - 10YR6/3 on the Munsell color chart). The lithological properties of this unit are characterized as a poorly sorted sediment with a Mz of 196 µm (Fig. 3a) that is comprised of: quartz grains, dark silty clay aggregates, 1 cm Glycymeris and Tucetona shells, 1.5 cm limestone pebbles, Gastropod Mollusk shells as well as Calcareous sandstone fragments which are notably found closer to its bottom (Fig. S4). F3 contains substantially lower Al/Si Ti/Si Zn/Si; Fe/Si, Rb/Si ratios compared to its underlying unit (F2) as well as MS values of ~50×SI-6 (Fig. 3a) and was dated in core D4 by one OSL age to 10.19 ± 0.90 ka (Tables 1, 2; Fig. 3a). Based on the sedimentological properties as well as the accompanying features unit F3 is interpreted as coastal/shallow marine sand.

4.2.5 Unit F4

Unit F4 unconformably overlies unit F3 and was identified in Boreholes D4, D12, D6. The surface elevations of this unit range from ˗2.2 to ˗3.2 PMSL and a thickness of 0.4 to 1.0 m (Figs. S1-S3). Brightness and color comprise of values of ~40 and dark grey – dark brown hues respectively (10YR3/1 - 10YR3/2 on the Munsell color chart). Lithological properties of this unit consist of a homogenous silty loam sediment with a Mz of 3 µm. Brackish microfauna, sea urchin spine remains, and gastropod shells were identified in the upper 0.3 of the unit (Dataset 1). The elemental ratios of Al/Si Ti/Si Zn/Si; Fe/Si, Rb/Si are considerably higher compared to its underlying unit (F3) and resemble those of F2. MS values range from 285×SI-6 in its lower parts and decrease upward until reaching 160×SI-6 in the surface of the unit (Fig. 3a).

Based on these petro-sedimentological properties as well as skeletal assemblage unit F4 was interpreted as a wetland deposit. The wetland facies bottom half was dated in core D4 by one OSL age to 9.15 ± 0.78 ka while its surface was dated in core D12 to 7.78 ± 0.71 ka (Tables 1, 2; Fig. 3b).

4.2.6 Unit F5

Unit F5 unconformably superimposes unit F1 and consists of a lower elemental composition of Al/Si Ti/Si Zn/Si; Fe/Si, Rb/Si ratios (Figs. S1-S3) as well as MS values that range from 2 to 12×SI-6 (Fig. 3) compared to the underlying unit F4. Altogether, based on the sedimentological characteristics, three facies were identified for unit F5:

1. Facies F5a was identified in Boreholes D4 and D12 with surface elevations of ˗1 PMSLD and thickness range of 1 – 2 m (Fig. 3a and S1-S3). Brightness and color consist of values of 65 to 70 with light brown – yellow hues respectively (10YR6/4 – 10YR6/3 on the Munsell color chart). Lithological properties are characterized as a homogenous sand sediment with 0.2 cm shell remains and a Mz of 200 µm. Based on these litho-sedimentological properties facies F5a was interpreted as aeolian sand dated in core D4 to 6.95 ± 0.68ka in its lowermost part and 6.56 ± 0.61 ka in its upper part (Table S3; Fig. 3a).

2. Facies F5b was identified in Boreholes D4, D12 and D6 with surface elevations of ˗0.8 to 0.3 PMSL and a thickness range of 0.4 to 1 m (Figs. 3a and S1-S3). Brightness and color consist of values of 50 – 90 and light grey – light yellow hues respectively (10YR7/4 – 10YR6/3 on the Munsell color chart). The sedimentological properties of this unit are characterized as coarse sand – gravel that includes pottery shards, iron pieces, chert remains, aeolianite fragments, limestone pebbles and bivalve shells. (Fig. 3). Based on these sedimentological properties, comprising anthropogenic features and OSL ages facies F5b was interpreted as coastal sand settlement horizons of the Iron Age – Early Muslim period.

3. Facies F5c was identified in Boreholes D4, D12 and D6 with surface elevations of 0.7 – 1 PMSL and thickness range of 1 – 1.5 m (Fig. 3a and Fig. S1-S3). Brightness and color consist of values of 50 – 70 and light grey – light brown hues respectively (10YR5/3 – 2.5YR6/6 on the Munsell color chart). The sedimentological properties of this unit are characterized as mid-size sand that includes bivalve shells and glass fragments in the upper 0.3 of the unit. (Fig. 3a). Based on these sedimentological properties and accompanying features facies F5c was interpreted as modern coastal sand.

<
