# Supplementary material for: A Neolithic mega-tsunami event in the eastern Mediterranean: Prehistoric settlement vulnerability along the Carmel coast, Israel
Source: PLoS One. 2020 Dec 23;15(12):e0243619. doi: 10.1371/journal.pone.0243619 (PMC7757801; doi:10.1371/journal.pone.0243619)
Supplement: S3 File — (DOCX) [file pone.0243619.s011.docx]

Link to Simulated model of the Tsunami event

A preliminary computer simulation of the Dor landslide and tsunami using the Tsunami Squares Method [26] indicates the possibility of a flow depth of 12 m (blue line) and run-ups of 16 - 20 m (red line). The end result of the model is presented in the figure below -


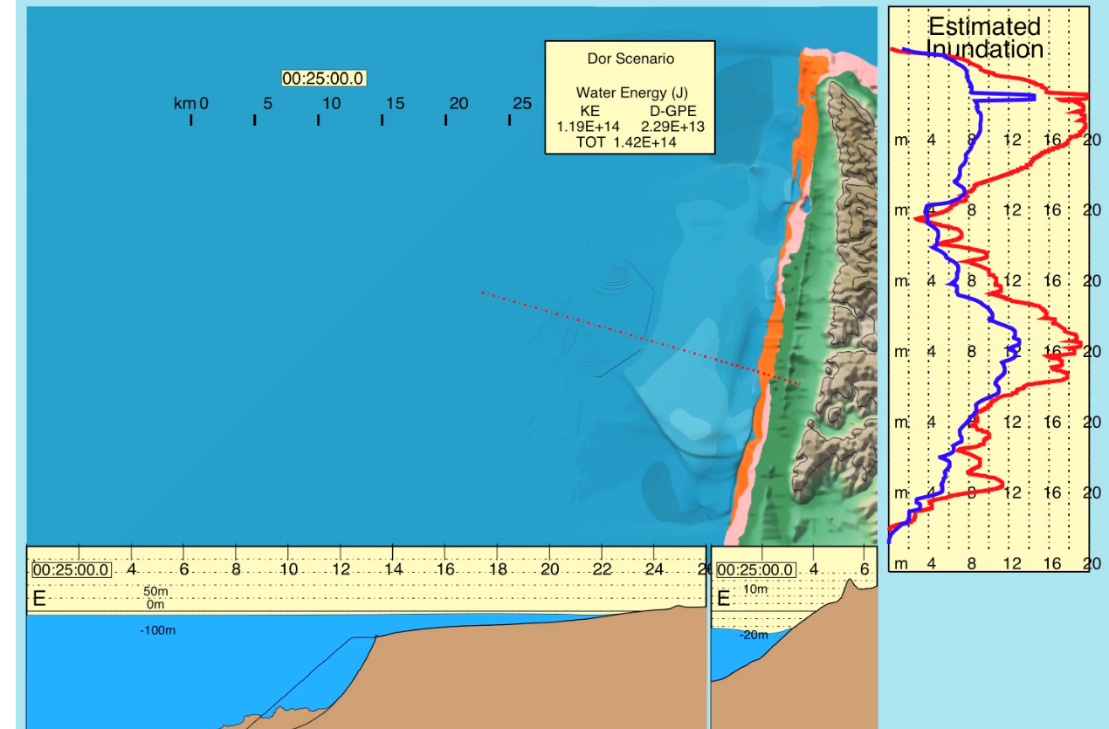


The following links present the various simulation runs:

Dor-slide

<https://websites.pmc.ucsc.edu/~ward/dor-slide-s.mov>

Dor-wave

<https://websites.pmc.ucsc.edu/~ward/dor-wave-s.mov>

Dor-wave view north

<https://websites.pmc.ucsc.edu/~ward/dor-wave-pn-s.mov>

Dor-wave with velocities.

<https://websites.pmc.ucsc.edu/~ward/dor-wave-v-s.mov>

Although this model shows the possibility of such a tsunamic occurrence for the lower age constrains (9,300 years ago – sea level elevations were around -16 m relative to PMSL) the validation of the model is beyond the scope of the current study and will be conducted in a follow-up investigation.
