# Supplementary material for: A Neolithic mega-tsunami event in the eastern Mediterranean: Prehistoric settlement vulnerability along the Carmel coast, Israel
Source: PLoS One. 2020 Dec 23;15(12):e0243619. doi: 10.1371/journal.pone.0243619 (PMC7757801; doi:10.1371/journal.pone.0243619)
Supplement: S4 File — (DOCX) [file pone.0243619.s012.docx]

**References**

1. M. P. Bernasconi, R. Melis, J.D. Stanley, Benthic biofacies to interpret Holocene environmental changes and human impact in Alexandria's Eastern Harbour, Egypt. The Holocene 16, 1163-1176 (2006).
2. G. A. Papadopoulos et al., Historical and pre-historical tsunamis in the Mediterranean and its connected seas: Geological signatures, generation mechanisms and coastal impacts. Mar. Geol. 354, 81-109 (2014).
3. D. Kelletat, G. Schellmann, Tsunamis on Cyprus: field evidences and 14C dating results. Z. Geomorphology. 46, 19-34 (2002).
4. E. G. Reinhardt et al., The tsunami of 13 December A.D. 115 and the destruction of Herod the Great's harbor at Caesarea Maritima, Israel. Geology 34, 1061-1064 (2006).
5. B. N. Goodman-Tchernov, H. W. Dey, E. G. Reinhardt, F. McCoy, Y. Mart, Tsunami waves generated by the Santorini eruption reached Eastern Mediterranean shores. Geology 37, 943-946 (2009).
6. B. N. Goodman-Tchernov, J. A. Austin, Deterioration of Israel's Caesarea Maritima's ancient harbor linked to repeated tsunami events identified in geophysical mapping of offshore stratigraphy. J. of Archaeol. Sci Rep. 3, 444-454 (2015).
7. S. Marco, O. Katz, Y. Dray, Historical sand injections on the Mediterranean shore of Israel: evidence for liquefaction hazard. Nat. Hazards 74, 1449-1459 (2014).
8. N. Tyuleneva, Y. Braun, T. Katz, I. Suchkov, B. Goodman-Tchernov,. A new chalcolithic-era tsunami event identified in the offshore sedimentary record of Jisr al-Zarka (Israel). Mar. Geol. 396, 67-78 (2018).
9. A. Scheffers, S. Scheffers, Tsunami deposits on the coastline of west Crete (Greece). Earth Planet. Sci. Lett. 259, 613-624 (2007).
10. B. Shaw et al., Eastern Mediterranean tectonics and tsunami hazard inferred from the AD 365 earthquake. Nature Geoscience 1(4), 268 (2008).
11. H.J. Bruins et al., Geoarchaeological tsunami deposits at Palaikastro (Crete) and the Late Minoan IA eruption of Santorini. J. of Archaeol Sci. 35, 191–212 (2008).
12. A. Scheffers, D. Kelletat, A. Vött, S. M. May, S. Scheffers, Late Holocene tsunami traces on the western and southern coastlines of the Peloponnesus (Greece). Earth Planet. Sci. Lett. 269, 271-279 (2008).
13. T. Willershäuser, A. Vött, H. Hadler, K. Ntageretzis, K. Emde, H. Bruckner, Holocene palaeotsunami imprints in the stratigraphical record and the coastal geomorphology of the Gialova Lagoon near Pylos (southwestern Peloponnese, Greece). Z. Geomorphol. 59 Supplement: 4. 215-252 (2015).
14. G. A. Papadopoulos, Tsunamis. Physical Geography of the Mediterranean, J. Woodward, ed. (Oxford University Press, 2009), pp 493–512.
15. G. A.Papadopoulos et al., Strong earthquakes and tsunamis in the East Hellenic arc. Research in Geophysics 2. 90–99 (2012).
16. K. Minoura et al., Discovery of Minoan tsunami deposits. Geology 28, 59–62 (2000).
17. A. Elias et al., Active thrusting offshore Mount Lebanon: source of the tsunamigenic AD 551 Beirut–Tripoli earthquake. Geology 35, 755–758 (2007).
18. A. Salamon, T.Rockwell, S.N.Ward, E.Guidoboni, A.Comastri, Tsunami hazard evaluation of the eastern Mediterranean: historical analysis and selected modeling. Bull. Seismol. Soc. Am. 97 (3), 705–724 (2007).
19. N. N. Ambraseys, Earthquakes in the Mediterranean and Middle East, A Multidisciplinary Study of Seismicity up to 1900. (Cambridge Univ. Press, Cambridge, UK 2009) p 947.
20. A. Fokaefs, G. A. Papadopoulos, Tsunami Hazard in the East Mediterenenean: Strong earthquicks and tsunamis in Cyprus and the Levant Sea. Natural Hazards 40(3), 503-526 (2007).
21. A. Salamon, T. Rockwell, E. Guidoboni, A. Comastri, A critical evaluation of tsunami records reported for the Levant coast from the second millennium BCE to the present, Israel. Isr. J. Earth Sci. 58 (3–4), 327–354 (2009).
22. I. Kuijt, N. Goring-Morris, Foraging, farming, and social complexity in the Pre-Pottery Neolithic of the southern Levant: a review and synthesis. Journal of World Prehistory 16, 361-440 (2002).
23. A. S. Murray, A. G. Wintle, The single aliquot regenerative dose protocol: potential for improvements in reliability, Radiat. Meas. 37, 377-381 (2003).
24. G. Guérin, N. Mercier, G. Adamiec, Dose-rate conversion factors: update. Ancient TL 29, 5-8 (2011).
25. M. J. Aitken, J. C. Alldred, The assessment of error limits in thermoluminescence dating. Archaeometry 14, 257-267 (1972).
26. J. Wang, S.N. Ward, L. Xiao, Tsunami Squares modeling of landslide generated impulsive waves and its application to the 1792 Unzen-Mayuyama mega-slide in Japan. Engineering Geology 256, 121-137 (2019) https://doi.org/10.1016/j.enggeo.2019.04.020.
