# Supplementary material for: A Neolithic mega-tsunami event in the eastern Mediterranean: Prehistoric settlement vulnerability along the Carmel coast, Israel
Source: PLoS One. 2020 Dec 23;15(12):e0243619. doi: 10.1371/journal.pone.0243619 (PMC7757801; doi:10.1371/journal.pone.0243619)
Supplement: Attachment — Submitted filename: Response to reviewers.docx [file pone.0243619.s013.docx]

Response to reviewers:

A Neolithic Mega-Tsunami Event in the Eastern Mediterranean: Prehistoric Settlement Vulnerability Along the Carmel Coast, Israel

Dear Prof. Peter F. Biehl and PLOS ONE editorial board,

We would like to thank you and the anonymous referees for the time and effort devoted to reviewing and improving our manuscript. We have accepted the majority of comments and suggestions and followed your instructions carefully while preparing this response document.

Below, we first present each editorial/reviewer comment raised by the referees, followed by the corrected text and /or specific responses. In order to distinguish between them, the reviewer comments are stated in red font, and our responses and edited texts are typed in blue.

The referees' comments and suggestions have led to the revision of the manuscript, and we hope you will find this version suitable for publication in PLOS ONE.

Sincerely,

Dr. Gilad Shtienberg – on behalf of the authors

Department of Anthropology/Scripps Center for Marine Archaeology

University of California San Diego
La Jolla CA 92093-0244

USA

Editorial comments

1. Please ensure that your manuscript meets PLOS ONE's style requirements, including those for file naming.

The required modification were done.

2. In your Methods section, please provide additional information regarding the permits you obtained to collect samples for the present study.

The permit information was added to the method section.

3. Please remove any funding-related text from the manuscript

The funding related text was deleted from the text of the manuscript

4. let us know how you would like to update your Funding Statement

Please update my funding statement.

'The Authors gratefully acknowledge the generous support provided by Scripps Center for Marine Archaeology, Scripps Institution of Oceanography, UC San Diego; The Koret Foundation (Grant ID 19-0295); Murray Galinson San Diego – Israel Initiative; the Israel Institute (Washington, D.C.); Marian Scheuer-Sofaer and Abraham Sofaer Foundation; Norma and Reuben Kershaw Family Foundation; Ellen Lehman and Charles Kennel - Alan G Lehman and Jane A Lehman Foundation; Paul and Margaret Meyer and the Israel Science Foundation (Grant ID 495/18).

5. Please complete your Competing Interests statement to state any Competing Interests

We declare that we the authors have declared that no competing interests exist.

6. We note that Figures 1, 2 and 6 in your submission contain map/satellite images which may be copyrighted.

As suggested by PLOS ONE editorial office the public domain natural earth data (https://www.naturalearthdata.com/downloads/50m-physical-vectors/50m-physical-labels/) was used for creating figure 1, and a relevet link was added to the figure caption.

Appropriate copyrights were acquired from the Geological Survey of Israel (please see correspondence below) for a 25 m raster grid that captures the surface elevation of the eastern Mediterranean (referenced from Hall et al. 1994) as well as shapefiles of lithology and steams for the Carmel coast.

These files were the basis for creating figure 2a by the corresponding author of the manuscript.

*Dear Gilad,*

*We are happy to provide explicit permission for use of data from the Geological Survey of Israel that was made in the two figures attached under the CC BY 4.0 license.*

*Please verify adequate citation of John Hall's data in your manuscript.*

*All the best,*

*Dr. Amit Mushkin*

*-----*

*Head, Geological Mapping Division*

*Geological Survey of Israel,*

*Jerusalem, Israel*

Figures 2b (digital elevation model; borehole location) and 6 (aerial photograph) were created in the current study using a drone and by implementing photogrammetry technics.

7. PLOS requires an ORCID iD for the corresponding author

Done, my ORCIS iD number is: 0000-0003-0303-7872.

Reviewer 1 comments

1. The use of time-scales 'ya' is non-standard and does not correspond to any known physical units of time measurements. Please replace by calBP. The same applies to the term 'years ago', for which the same applies. Both 'ya' and 'years ago' sound rather home-made.

The use of calBP is reserved for radiocarbon dating, and since our radiometric dating technique relies solely on optically stimulated luminance, we used ka (kilo-annum) (Nelson et al., 2015) throughout the revised manuscript and figures.

2. Your use in the Titel of the buzzword 'resilience' contradicts your statement in the Conclusion that tsunamis played a 'destabilizing role'. Either 'resilience' or 'destabilizing': Tertium non Datur. Looking closer, it becomes clear that you are differentiating between two periods, one (early Holocene) that has a lot of tsunamis, and the other (later Holocene) that has few tsunamis. A very nice result ! But, what this to do with 'resilience' or' destabilization'? IF it is true – as you write – that the early Holocene PPNA-B settlements were mega-tsunami-destroyed, how can you know whether the corresponding societies were 'tsunami-destabilized'? Similarly, IF it is true - as you write – that the many later PPNB-C sites indicate resettlement following the mega-tsunami, what can this have to do with 'tsunami-resilience'? Does not the whole question boil down to what we know - or do not know - about societal reactions towards adverse conditions (whether fast or slow, whether climatic and/or society-internal). Of course, it is not your task to resolve these questions ! Even so, I think that the very word resilience is not particularly convincing , when applied to potential tsumami impact and societal consequences.

After carefully examining this comment, we agree with Reviewer 1 and replaced the word Resilience with Vulnerability in the title.

3. Reference Line 386: you write: Recovery from the early Holocene tsunami event was slow, possibly also affected by the 9,250 ya cold and arid climatic event [49]. But: reference [49] does not find evidence for impact of climate on prehistoric societies ! According to [49], prehistoric societies are resilient towards climate variability.

We agree with the statement of Reviewer 1 and erased the sentence, "possibly also affected by the 9.25 ka cold and arid climatic event [49]" from the manuscript.

4. The following sentence is both misplaced and not understandable: Line 376: By the PPNA and PPNB, the heartland of prehistoric settlement had shifted to the interior of the southern Levant [42,48]. True is (& perhaps you mean ?): the (well-known) major expansion both in size of sites as well as settlement area (call it 'across the Jordan from West to East) at around 9.3 ka calBP,

According to the comment raised by Reviewer 1, we changed the sentences as follows:

The rapid growth in size and area of autonomous Middle PPNB settlements in the hill country of Israel and across the Jordan area may reflect the move away from the coastal lowlands [47,48].

Reviewer 2 comments

1. Does the proposed paleotsunami evidence indicate such a devastating impact that the entirety of ca. 4000 years of settlement evidence would have been completely destroyed? Perhaps one might expect even ephemeral traces of remaining architecture (slight though such architecture may have been in the LN/PPNA), or some surviving skeletal evidence (e.g. complete animal and/or human skeletons which might indicate the sudden impact of the tsunami; or injury evidence, and so on). Evidence for the lack of Late/Final Natufian and PPNA sites (which is not uncommon throughout the southern Levant at this time) could also be due to landscape/settlement practices by human communities, or to archaeological survey biases. All these factors would have to be discounted before a firm conclusion based on such a very localized event could be substantiated.

The issue of the identification of paleo-tsunami deposits in the geological record, as well as its possible impact on the landscape and ancient societies, is a highly charged issue in the earth and social sciences. Accordingly, we began our research on this issue with great skepticism. From the time we extracted the cores in the field in Israel in July 2018 to the analyses of the cores here at UCSD – SIO, we have been very cautious in our interpretation, only finalizing the results after obtaining the radiometric dates of the poorly sorted quartz sand layer that consists of marine mollusk shells limestone pebbles, calcareous sandstone clasts, and dark, silty clay rip-up clasts as well as the overlying and underlying units. Once we were confident with our interpretation, we used the analyses of the depositional patterns in the Dor sediment cores to provide insights for identifying how the ancient tsunami impacted the prehistoric settlement patterns of the Carmel coast.

We raise our hypothesis regarding the impact of the paleo-tsunami on coastal societies after carefully considering the possible outcomes of such a devastating event on the low-lying areas located a few kilometers from the present shoreline. The consequence of such a tsunami may have dramatically reduced the carrying capacity of the Carmel coast's lowland areas by increasing the salt concentrations of the soils and underlying aquifer and destroying fields, pasture, and herds leading to significant economic disruption.

The strong catastrophic event is also believed, similar to modern-day incidents, to have had a profound impact on the landscape, eroding a few tens of centimeters from the paleo-surface (Chagué-Goff et al., 2011; Paris et al., 2009; Yamada et al., 2014). This known phenomenon could explain why numerous expeditions that have taken place over the last decades in the shallow marine and coastal parts of the Carmel coast (Barkai and Biran, 2012; Edwards, 2016; Galili et al., 2019; Galili et al., 1993; Galili and Nir, 1993; Galili et al., 1997; Galili and Weinstein-Evron, 1985; Goring-Morris, 1984; Meier et al., 2017) have not found Natufian – Pre pottery Neolithic B sites nor human remains from the lowlands while the existence of such sites has been identified in the Carmel coast in areas with elevations that are higher than 20 m with respect to present-day mean sea level. We hope this addresses the query of Reviewer 2.

Reviewer 3 comments

1. The large range of the dating, so that it is difficult to be sure about the synchronicity between the tsunami and described changes in settlement patterns (e.g. the tsunami might have occurred only after the inland MPPNB sites grew; or it might have occurred only shortly before the LPPNB so that the impact was not long term.

Please refer to our response that answered comment 1 raised by Reviewer 2.

2. The area actually reconstructed to be destroyed, 1.5-3.5km inland in one area of the coast, relatively to the rest of the 'Neolithic' area. As such I would argue it goes too far to claim that the event would have caused "a reversal of the Neolithic Demographic Transition" (line 373) or to imply that absence of tsunami events allowed for "unhindered growth" afterwards.

We agree with this comment and changed the text accordingly:

"With carrying capacity and fertility decreased, population mobility is thought to increase, influencing the Neolithic demographic transition and possibly decreasing population density".

.

3. Related to the above, I would also like to see more on where the site pattern information is based on. For example in Figure 2 it is very useful that submerged PPNC and PN sites are indicated, and in addition it would be very useful to see also the other, inland known sites by period. Currently five such sites are indicated but not coded by period, and it would be good to know if, and where, other sites of the period are within the map area (or, for which area on the map the 'complete' settlement pattern is given). Perhaps this could be a separate figure if this one gets too busy otherwise. In addition, it would be good to briefly discuss the background of the settlement pattern, and mainly if it could be affected by a research bias (e.g. a large research project focusing on a specific sub-area, or extensive building work and therefore rescue archaeology in certain places) or not. It is no problem if this turns out to be the case, it just needs to be taken into account.

Considering comment 3 we have color-coded the known terrestrial Natufian – PPNC sites in figure 2 and made the necessary changes in the figure caption and text.

4. Consistency in dates: Throughout the paper the way dates are presented should be made consistent. Currently yr, ka yr, years ago, calibrated years before present, and BC/AD (Supplementary Tables 1+2) are all used, which makes it confusing to the reader. The dating used for the archaeological periods in the Supplementary Information should also be the same as in the main text and throughout the text.

In accordance with comment four, we have changed the temporal unit for the OSL ages to ka (kilo-annum) in the text, tables, and figures. Additionally, we have added the temporal unit ka to the ^14^C calibrated dates to text and figures.

5. Not so much rare in general, but for tsunami events I agree, although it might be worth citing some studies that have looked at this (e.g. Waddington and Wicks 2017 Journal of Archaeological Science).

The word "rare" was changed into "less common," and the reference was added to the manuscript.

6. Figure 1 and caption: it would be useful to have the names of the numbered sites here in the figure or in the caption, not only in the Supplementary Information.

In accordance with comment 6 we added the site names to the figure caption.

7. Line 107-109: I would probably move this sentence to the Materials and Methods section and instead add here a sentence specifically stating the aims/objectives of the study.

The last sentence found in the introduction was removed from the text, and as Reviewer 3 suggested, the main objective of this study was added:

"The preservation of Quaternary deposits and landforms at Dor coupled with the abundance archaeological sites provide a unique opportunity to investigate the geomorphological processes and human-landscape interactions in the eastern Mediterranean".

8. OSL dating: Was an age model done? If not, why not? And if yes, how was it done?

Equivalent Dose (D_E_) was calculated for each OSL age using the central age model (CAM) for all samples. Aliquots were rejected if they had evidence of feldspar contamination, recycling ratio <0.1 or >1.1, recuperation >10% of the natural signal, or natural DE greater than the highest regenerative dose given. Errors on DE values are reported at 2-sigma standard error, and age estimates are reported at 1-sigma standard error (see Table 1). Uncertainties include errors related to instrument calibration, dose rate, and equivalent dose calculations and calculated in quadrature (Aitken, 2003; Guérin et al., 2011). This explanation is presented in the extended methods section found in the supporting information file.

9. Table 1: Please present the dates in a consistent way. It says in the caption they are in ka years, but in the table they are partly in years and partly in ka year.

The units for time measurements presented in the manuscript figure and tables are all presented in a similar - ka (kilo-annum).

10. Table 2: This could perhaps go in the Supplementary Information.

The research that is presented in our manuscript relies on the OSL ages that we have generated in our study. Because these ages are such an essential part of the investigation, we think that both Tables 1 and 2, which present the optically stimulated measurements, are needed in the main text and will benefit the readers.

11. Lines 219-221: repetition, already stated in the Methods section

This line was erased from the manuscript.

12. Line 424: "earliest" I suggest "earliest known", there are probably earlier, yet undocumented ones

The word "known" was added to the sentence.

13. What is the difference between Table S1 and S2? Why are these not in one table?

These two tables present a compilation of prehistoric - historic tsunami deposits (Table S1) and documented Tsunami events (Table S2) from the eastern Mediterranean. And thus, we think that should be kept separate.

14. Remove hyphens between adjectives and nouns, e.g. eastern-Mediterranean should be eastern (or Eastern) Mediterranean, early-Holocene should be early Holocene, marine-sand should be marine sand.

These grammar mistakes were amended.

15. Line 77: "Less" should be with lower case

This spelling error was corrected.

16. "Suplement matirial" should be "Supplemental material" (or as called in PLOS ONE "Supporting Information").

The name of the file was changed accordingly.

References

Aitken, M., 2003. Radiocarbon dating. Archaeological Method and Theory, 505-508.

Barkai, R., Biran, N., 2012. Aviel: a new Neolithic site at the foothills of Mt. Carmel. NEO-LITHICS 2/11 14.

Chagué-Goff, C., Schneider, J.-L., Goff, J.R., Dominey-Howes, D., Strotz, L., 2011. Expanding the proxy toolkit to help identify past events — Lessons from the 2004 Indian Ocean Tsunami and the 2009 South Pacific Tsunami. Earth-Science Reviews 107, 107-122.

Edwards, P.C., 2016. The chronology and dispersal of the Pre-Pottery Neolithic B cultural complex in the Levant. Paléorient, 53-72 %@ 0153-9345.

Galili, E., Benjamin, J., Eshed, V., Rosen, B., McCarthy, J., Kolska Horwitz, L., 2019. A submerged 7000-year-old village and seawall demonstrate earliest known coastal defence against sea-level rise. PLoS One 14, e0222560.

Galili, E., Dahari, U., Sharvit, J., 1993. Underwater surveys and rescue excavations along the Israeli coast. The International Journal of Nautical Archaeology 22, 61-77.

Galili, E., Nir, Y., 1993. The submerged Pre-Pottery Neolithic water well ofAtlit-Yam, northern Israel and its palaeoenviromental implications. The Holocene 3, 265-270.

Galili, E., Stanley, D.J., Sharvit, Y., Weinstein‐Evron, M., 1997. Evidence for Earliest Olive-Oil Production in Submerged settlments off the Carmel Coast, Israel. Tournal of archaeologica Science 24, 1141-1150.

Galili, E., Weinstein-Evron, M., 1985. Prehistory and paleoenvironments of submerged sites along the Carmel coast of Israel. Paleorient 11, 37-52.

Goring-Morris, A.N., 1984. The lithic assemblages from Tel Mevorakh. Excavations at Tel Mevorakh (1973-1976), Qedem 18, 81-86.

Guérin, G., Mercier, N., Adamiec, G., 2011. Dose-rate conversion factors: update. Ancient TL 29, 5-8.

Meier, J.S., Goring-Morris, A.N., Munro, N.D., 2017. Aurochs bone deposits at Kfar HaHoresh and the southern Levant across the agricultural transition. antiquity 91, 1469-1483 %@ 0003-1598X.

Nelson, M.S., Gray, H.J., Johnson, J.A., Rittenour, T.M., Feathers, J.K., Mahan, S.A., 2015. User Guide for Luminescence Sampling in Archaeological and Geological Contexts. Advances in Archaeological Practice 3, 166-177.

Paris, R., Wassmer, P., Sartohadi, J., Lavigne, F., Barthomeuf, B., Desgages, E., Grancher, D., Baumert, P., Vautier, F., Brunstein, D., Gomez, C., 2009. Tsunamis as geomorphic crises: Lessons from the December 26, 2004 tsunami in Lhok Nga, West Banda Aceh (Sumatra, Indonesia). Geomorphology 104, 59-72.

Yamada, M., Fujino, S., Goto, K., 2014. Deposition of sediments of diverse sizes by the 2011 Tohoku-oki tsunami at Miyako City, Japan. Marine Geology 358, 67-78.
